# Note on extremal problems about connected subgraph sums

**DOI:** 10.1007/s00373-026-03026-8

**Published:** 2026-03-06

**Authors:** Stijn Cambie, Carla Groenland

**Affiliations:** 1 Department of Computer Science, KU Leuven Campus Kulak-Kortrijk, 8500 Kortrijk, Belgium; 2 Delft Institute of Applied Mathematics, TU Delft, Delft, Netherlands

**Keywords:** Connected subgraph sums, Sidon set, Golomb ruler, Graph reconstruction, Graph labelling, 05D99, 05C78, 05C99

## Abstract

For a graph *G* with vertex assignment $c:V(G)\rightarrow \mathbb {Z}^+$, we define $\sum _{v\in V(H)}c(v)$ for a connected subgraph *H* of *G* as a connected subgraph sum of *G*. We study the set *S*(*G*, *c*) of connected subgraph sums and, in particular, resolve a problem posed by O.-H. S. Lo in a strong form. We show that for each *n*-vertex graph *G*, there is a vertex assignment $c:V(G)\rightarrow \{1,\dots ,12n^2\}$ such that for every *n*-vertex graph $G'\not \cong G$ and vertex assignment $c'$ for $G'$, the corresponding collections of connected subgraph sums are different (i.e., $S(G,c)\ne S(G',c')$). We also provide some remarks on vertex assignments of a graph *G* for which all connected subgraph sums are different.

## Introduction

For a graph *G*, a subgraph $H \subseteq G$ and a vertex assignment $c :V(G) \rightarrow \mathbb Z^+$ with $\mathbb {Z}^+=\{1,2,3,\dots \}$, we denote $c(H)=\sum _{v \in V(H)} c(v)$ as the *subgraph sum* of *H*. The set

$$ S(G,c)= \{c(H) \mid H {\text { connected subgraph of }}G\} $$

is the set of all subgraph sums over all connected (induced) subgraphs of *G* for vertex assignment *c*. Here we do not consider the empty graph as a subgraph (this convention has no impact on the results, besides *S*(*G*, *c*) containing 0 or not).

For example, when $G=K_n$ has vertex set $\{1,\dots ,n\}$, then

$$ S(K_n,c)= \left\{ \sum _{i\in U}c(i): U\subseteq \{1,\dots ,n\}\right\} $$

is the collection of subset sums of the positive integers $c(1),\dots ,c(n)$.

For a tree *T*, *S*(*T*, *c*) is also called the tree spectrum [6]. The study of the tree spectrum in [6] was inspired by a connection to the cycle spectrum of planar Hamiltonian graphs. On-Hei Solomon Lo [6] studied the number of values in a certain range (interval) belonging to *S*(*T*, *c*). Relatedly, one can remark that no more than $$\left( {\begin{array}{c}n\\ \lfloor \frac{n}{2}\rfloor \end{array}}\right) $$ many values can appear in *S*(*G*, *c*) for interval lengths smaller than $\min _{v \in V(G)} c(v)$, by the Littlewood-Offord theorem or Sperner’s theorem. The latter is still true when considering the set *S*(*G*, *c*) as a multiset.

It is natural to expect that *G* has some influence on which *S*(*G*, *c*) are possible (for different choices of *c*) and that this may depend on the size of the “vertex weights” that are allowed. For a graph *G* and positive integer *N*, let $S(G;N)=\{S(G,c)\mid c:V(G)\rightarrow \mathbb {Z}^+ {\text { with }}c(G)=N\}.$ Lo [6] (at the end of the conclusion section) made the following conjecture.

### Conjecture 1

For any two non-isomorphic connected graphs $G_1$ and $G_2$ on *n* vertices, $S(G_1; 2^n-1) \not = S(G_2; 2^n-1).$

We prove this conjecture and strengthen it in two directions.

Firstly, we show that it is even possible to select an element in $S(G;2^n-1)$ that is unique to *G*.

### Theorem 1

Let *G* be a connected graph on *n* vertices. Then there exists $c:V(G)\rightarrow \{1,\dots ,2^n-1\}$ with $c(G)=2^n-1$ such that for any *n*-vertex graph $G'$, if there is a vertex assignment $c':V(G)\rightarrow \mathbb {Z}^+$ satisfying $S(G,c) = S(G',c')$, then $G\cong G'$.

Here we assign the powers of 2 from 1 up to $2^{n-1}$, and connectedness implies $c(G)=2^n-1$. The result above immediately implies the conjecture. For large *n*, we also reduce $2^n-1$ to $12n^3$.

### Theorem 2

Let *G* be a graph on *n* vertices. Then there exists $c:V(G)\rightarrow \{1,\dots ,12n^2\}$ such that $S(G,c)=S(G',c')$ such that for any *n*-vertex graph $G'$, if there is a vertex assignment $c':V(G)\rightarrow \mathbb {Z}^+$ satisfying $S(G,c) = S(G',c')$, then $G\cong G'$. When *G* is connected, the assumption that $G'$ has *n* vertices may be replaced by the assumption that $G'$ is connected.

In other words, we can “reconstruct” *G* from knowing that there is a vertex assignment $c:V(G)\rightarrow \mathbb {Z}^+$ with a particular set of connected subgraph sums *S*(*G*, *c*), and only “modest” weights on the vertices are required for *c*, i.e., $c(G)=O(n^3).$ There is a wide literature on graph reconstruction questions, perhaps the most similar to ours being the reconstruction from the set of vertex sets of size *k* that induce a connected subgraph [2, 5].

Note that for any graph *G* and assignment *c*, the graph $G'=G+G$ obtained by taking the disjoint union of two copies of *G*, with $c'$ defined as *c* on both copies, has $S(G,c)=S(G',c')$. So some assumption of connectivity or on the number of vertices is required in Theorem 2.

The proofs of our main results are presented in Section 2. We make some observations about vertex assignments for which all connected subgraphs yield distinct sums in Section 3, which are related to the Erdős distinct subset sum problem, which asks about the minimum of $\max T$ among the sets $$T \in \left( {\begin{array}{c}\mathbb Z^+\\ k\end{array}}\right) $$ with distinct subset sums, and optimal Golomb rulers. In Section 4, we discuss potential directions for future work.

## Reconstruction from collection of connected subgraph sums

We show the following result which immediately implies Theorem 1.

### Lemma 1

Let $G=(V,E)$ be a connected graph with $V=\{v_1,\dots ,v_n\}$.

Let $c(v_i)=2^{i-1}$ for $i\in [n]$. Then $G\cong G'$ for every *n*-vertex graph $G'$ and $c':V(G)\rightarrow \mathbb {Z}^+$ with $S(G,c)=S(G',c')$.

### Proof

We order the vertices $w_1,\dots ,w_n$ of $G'$ such that $c'(w_1)\le \dots \le c'(w_n)$.

We first show by induction on *i* that $c'(w_i)\le 2^{i-1}$. This is true for $i=1$ because $1=c(v_1)\in S(G,c)=S(G',c')$ and $c'$ only assigns positive integer values. Assuming the claim holds for $w_1,\dots ,w_j$, this implies that $\sum _{i=1}^j c'(w_j)<2^{j}$. So the value $2^j\in S(G',c')$ must be coming from a connected subgraph with a vertex that is not among $w_1,\dots ,w_j$ and therefore $c'(w_{j+1})\le 2^j$.

Next, since $2^n-1\in S(G,c)=S(G',c')$, we find that $c'(w_{j})=2^{j-1}$ for all *j*. This implies that $f:V(G)\rightarrow V(G')$ with $f(v_j)=w_j$ is an isomorphism since $v_iv_j\in E(G)$ if and only if $2^{i-1}+2^{j-1}\in S(G_1,c)=S(G_2,c')$ if and only if $w_iw_j\in E(G')$. $\square$

**Fig. 1:**
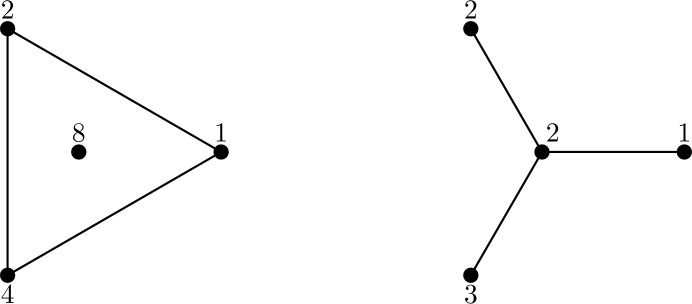
The graph *G* on the left with depicted weight assignment *c* and the graph $G'$ on the right with depicted weight assignment $c'$ satisfy $S(G,c)=\{1,2,3,4,5,6,7,8\}=S(G,c')$

As shown in Figure 1, we cannot simply remove the assumption that *G* is connected from the statement of Lemma 1.

The example $S(K_n,c)=\{1,\ldots ,2^n-1\}=S(P_{2^n-1},c')$ where $c(v_i)=2^{i-1}$ and $c' \equiv 1$, indicates that the graphs *G* and $G'$ having the same order is a necessary restriction as well.

Next, we prove the stronger bound of $c(G)\le 12n^3$ (where furthermore connectedness is not needed).

### Proof of Theorem 2

Let *G* be a graph on *n* vertices. We want to show that there exists $c:V(G)\rightarrow \{1,\dots ,12n^2\}$ such that $S(G,c)=S(G',c')$ implies that $G\cong G'$.

The claim below follows from the Sidon set construction from Erdős and Turán [4], but we include the proof for convenience to get the exact statement we use. $\square$

### Claim

There exists a set $S=\{s_1, s_2, \ldots , s_n\}\subseteq \{1,\dots , 4n^2\}$, such that $s_i+s_{\ell }=s_j+s_k$ implies that $\{i,\ell \}=\{j,k\}$.

### Proof

By Bertrand’s postulate, there exists an odd prime $n+1\le p\le 2n-1$. For an integer *x*, let $r_p(x)\in \{0,\dots ,p-1\}$ denote the remainder of *x* after division by *p*. Set

$$ s_i=2pi + r_p(i^2) $$

for every $i \in [n]$. Now $s_i+s_\ell =s_j+s_k$ implies

$$ 2(i-j)p+ r_p(i^2)-r_p(j^2) = s_i-s_j=s_k-s_{\ell }=2(k-\ell )p+ r_p(k^2)-r_p(\ell ^2). $$

This implies that $i-j=k-\ell$ and $r_p(i^2)-r_p(j^2)=r_p(k^2)-r_p(\ell ^2)$. We are done if $i-j=0$, so suppose $i\ne j$. Since $p\ge n+1$, it follows that $i-j\not \equiv 0 \bmod p$. Since $(i+j)(i-j)=i^2-j^2\equiv k^2-\ell ^2\equiv (k+\ell )(k -\ell )\equiv (k +\ell )(i-j)\bmod p$, this shows that $i+j\equiv k+\ell \bmod p$, which together with $i-j=k-\ell$ implies that $i+j\equiv k+\ell \bmod {2p}$, which then using $p\ge n+1$ again shows that $i+j=k+\ell$. This now implies that $i=k$ and $j=\ell$. Note also that for all *i*, $s_i\le 2pi+(p-1)\le 2(2n-1)n+2n\le 4n^2$. $\square$

Let $V(G)=\{v_1, \ldots , v_n\}$. Let $M= 4n^2$. Let $1\le s_1\le \dots \le s_n\le M$ be the values from the claim above and set $c(v_i)=2M+s_i$ for every $i \in [n]$. Then $c(G)\le 3nM \le 12n^3$ and $c(v_1)\le \dots \le c(v_n)$.

Let $G'$ and $c'$ be given such that $S(G,c)=S(G',c')$. We will show that $G\cong G'$. Let $V(G')=\{w_1,\dots ,w_{n'}\}$. Since $2c(v_1)\ge 4M>3M\ge c(v_n)$, it follows that the *n* smallest weights in $S(G,c)=S(G',c')$ must be $c(v_1),\dots ,c(v_n)$ and $c'(w_1),\dots ,c'(w_{n})$ (up to relabeling of the vertices). So $c(v_i)=c'(w_i)$ for all $i\in [n]$. If *G* and $G'$ are both connected, then $c(v_1)+\dots +c(v_n)$ and $c'(w_1)+\dots +c'(w_{n'})$ both equal the largest value in $S(G,c)=S(G',c')$ and therefore $n=n'$ now follows.

Next, we use that $3c(v_1)\ge 3(2M+1)>2(2M+M)\ge 2c(v_n)$. This shows that all values in $\{4M,4M+1,\dots ,6M\}\cap S(G,c)$ are created via connected vertex sets of size 2 (also known as edges) in both *G* and $G'$. By construction, $c(v_i)+c(v_j)=c(v_k)+c(v_\ell )$ implies $s_i+s_j=s_k+s_\ell$ which implies $\{i,j\}=\{k,\ell \}$ (by choice of *S*). This means that $v_iv_j\in E(G)$ if and only if $4M+s_i+s_j\in S(G,c)=S(G',c')$ if and only if $w_iw_j\in E(G')$. The map which sends $v_i$ to $w_i$ for all $i\in [n]$ gives the desired isomorphism $G \cong G'$.

## Subgraph sum-distinct assignments

The set *S*(*G*, *c*) could contain values which appeared as the subgraph sum of multiple subgraphs. This is not the case for what we define as an SSD assignment *c*.

### Definition 1

Let $G=(V,E)$ be a graph. A *subgraph sum-distinct* (SSD) assignment *c* is a mapping $c :V \rightarrow \mathbb Z^+$ for which every two distinct connected induced subgraphs $G_1$ and $G_2$ satisfy $c(G_1)\not =c(G_2)$.

A natural question that arises is to determine optimal choices for *c*. The following two optimality criteria for *c* seem natural. Let $M(G)=\min _c \sum _{v \in V} c(v)$, where the minimum is taken over all possible SSD assignments *c*. Let $m(G)=\min _c \max \{ c(v) \mid v \in V\}$, where the minimum is taken over all possible SSD assignments *c*.

Specific cases of this have already been studied in the literature. For example, estimating $m(K_n)$ corresponds with the Erdős distinct subset sums problem [3]. We note that $M(K_n)=2^n-1$. Axenovich, Caro and Yuster [1] also studied a generalisation of *m*(*G*), where the vertices of a hypergraph are assigned positive integer weights and all hyperedges need to receive distinct sums; our setting is a special case of this where the hyperedges correspond to the connected subgraphs of a fixed graph. It follows from their result that $m(G)=o(k^2)$ if *G* is an *n*-vertex connected graph with $k=n^{O(1)}$ connected subsets.

For $G=P_n$, $M(P_n)$ is related to Golomb rulers or Sidon sets. Recall that a subset $S\subseteq \{1,\dots ,N\}$ is called a Sidon set if $x+y=z+w$ for $x,y,z,w\in S$ implies $\{x,y\}=\{z,w\}$.

### Proposition 1

$M(P_n)$ is the smallest integer *N* such that there is a Sidon set $S\subseteq \{1,\dots ,N\}$ of size *n*.

### Proof

Let *c* be an SSD assignment of $P_n=(V,E)$, where $V=\{v_1, v_2, \ldots , v_n\}.$ If $s_k=\sum _{i=1}^kc(v_i)$, then $s_k-s_j=\sum _{i=j+1}^k c(v_i)$, so the condition that all subpath sums are distinct is the same as the condition that all differences $s_k-s_i$ are distinct. Thus $\{s_1,\dots ,s_n\}$ is a Sidon set.

Conversely, a Sidon set $\{s_1,\dots ,s_n\}\subseteq \{1,\dots ,N\}$ gives rise to an SSD assignment *c* of $P_n$ with $c(P_n)\le N$, by inductively defining $c(v_1),\dots ,c(v_n)$ such that $\sum _{i=1}^jc(v_i)=s_j$. $\square$

**Fig. 2:**
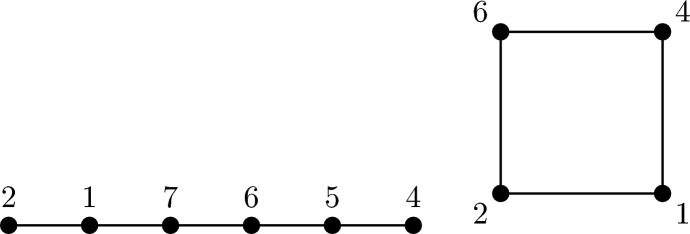
SSD assignments attaining *m*(*G*) and *M*(*G*) for $G \in \{P_6,C_4\}$

Similarly, the value $M(C_n)$ is exactly equal to the length of optimal circular Golomb rulers. Examples of (circular) Golomb rulers are presented in Figure2.

Of course, when *H* is a subgraph of *G*, then $m(H)\le m(G)$ and $M(H)\le M(G)$ since the assignment used for *G* can also be used for *H*. We also observe that, up to multiplicative constant, determining *m*(*G*) for $G=S_{n+1}=K_{1,n}$ the star on $n+1$ vertices is the Erdős distinct subset sum problem, and that $m(C_n)$ and $m(P_n)$ also have the same order.

### Proposition 2

$$m(K_n) \le m(S_{n+1}) \le 2m(K_n)$$ and $$m(P_n) \le m(C_n) \le 2m(P_{n-1})\le 2m(P_{n})$$.

### Proof

To see that $m(K_n)\le m(S_{n+1})$, note that every subset among the *n* leaves of the star must have a unique sum (after adding the center of the star to form a connected subset).

For $m(S_{n+1}) \le 2m(K_n)$, it is sufficient to double the values of an optimal assignment of $K_n$, assign those values to the leaves and assign a small odd number (e.g. 1) to the center.

The inequalities $m(P_n) \le m(C_n)$ and $m(P_{n-1})\le m(P_{n})$ are immediate, using that if *H* is a connected subgraph of *G*, then $m(H) \le m(G).$

To see that $m(C_n)\le 2m(P_{n-1})$, we double the values of an optimal assignment of (the induced) $P_{n-1}$ and assign a (small) odd number to the remaining vertex, *v*, of the $C_n$. Since the subgraphs can be divided in complementary pairs (their vertex sets partition $V(C_n)$), the assignment satisfies the conditions by definition. For this, note that the subgraphs not containing *v* are different even sums, and the subgraphs containing *v* are all odd (and different being the complements with respect to the total sum). $\square$

As a generalisation of the Erdős distinct subset sums problem and Sidon sets, one can wonder about the values of *m*(*G*) or *M*(*G*) for a certain graph or the extremes within a specified graph class. It is unclear to the authors if there are interesting graphs *G* for these parameters which are not $P_n$ or $K_n.$

## Conclusion

The conjecture of Lo [6] gives rise to the following question: Given *n*, what is the smallest value of *N* such that for any two non-isomorphic graphs *G* and $G'$ on *n* vertices, $S(G;N)\ne S(G';N)$? We showed that $N\le 12n^3$ in Theorem 2 but it is possible that the correct order of growth is quadratic in *n*. In Lo’s setting, it is allowed that $S(G;N)\subset S(G';N)$. If we want to avoid this, then the cubic bound that we prove is optimal. Indeed, let $G=K_n$ and $G'=K_n^-$, a clique minus one edge. Let $c:V(G) \rightarrow \mathbb Z^+$ with $c(G)=N$ for some $N<\frac{n^3}{100}$. Then there are at least 0.9*n* vertices which are assigned a value below $\frac{n^2}{10}$. Since $$\left( {\begin{array}{c}0.9n\\ 2\end{array}}\right) >2\frac{n^2}{10},$$ there are at least two pairs of vertices with the same sum. In particular, $G=K_n$ has two edges $e_1, e_2$ with $c(e_1)=c(e_2)$. We define $c'\in S(G';N)$ using *c* and the isomorphism $G'\cong K_n\setminus e_1$. Now $S(G,c)=S(G',c')$ since the only disconnected vertex subset in $K_n \setminus e_1$ is $e_1$, but its sum is also achieved by $e_2$.

We introduced the stronger variant in which we wish to find a vertex assignment *c* such that *S*(*G*, *c*) is unique to *G* in the sense that for any other graph $G'$ on the same number of vertices, if $S(G,c)=S(G',c')$ for some $c'$, then $G\cong G'$. By the example above and Theorem 2, for *n*-vertex graphs, the smallest total vertex weight is of order $\varTheta (n^3)$. When restricting both *G* and $G'$ to trees, we expect that a better bound may be possible.

### Question 1

Can Theorem 2 be improved when restricting to trees, in such a way that *c*(*G*) is $o(n^3)$ or even $O(n^{2})$?

## Data Availability

All relevant data generated or analyzed within this research is included in this article.
